# Oncologists’ Attitudes Toward Cancer Care Affordability

**DOI:** 10.1001/jamanetworkopen.2022.7863

**Published:** 2022-04-19

**Authors:** Emeline M. Aviki, Nadeem R. Abu-Rustum, Bridgette Thom, Haley A. Moss, Fumiko Chino

**Affiliations:** 1 Gynecology Service, Department of Surgery, Memorial Sloan Kettering Cancer Center, New York, New York; 2 Weill Medical College of Cornell University, New York, New York; 3 Affordability Working Group, Memorial Sloan Kettering Cancer Center, New York, New York; 4 Department of Medicine, Memorial Sloan Kettering Cancer Center, New York, New York; 5 Department of Obstetrics and Gynecology, Duke University Hospital, Durham, North Carolina; 6 Department of Radiation Oncology, Memorial Sloan Kettering Cancer Center, New York, New York

## Abstract

This investigator-designed survey study evaluates oncologists’ attitudes about cancer treatment affordability for patients and acceptability of physician-based solutions.

## Introduction

Patients with cancer face substantial financial hardship from the costs of treatment. More than 40% of patients with cancer have depleted their life’s savings within 2 years of diagnosis.^1^ This financial toxicity of treatment is managed reactively, at best, by referrals to financial assistance, philanthropy groups, and self-funding through online fundraisers.^2^

Physicians have varying perceptions about the extent of their role in addressing treatment affordability issues for the patient, and few feel comfortable initiating cost conversations.^3,4,5^ We evaluated oncologists’ attitudes on treatment affordability and the acceptability of physician-based solutions.

## Methods

In this survey study, a 16-item investigator-designed questionnaire was emailed to attending oncologists at a single academic cancer center on February 3, 2020. The survey closed on March 9, 2020. Participation was voluntary and uncompensated. Respondents reported only their sex (male or female) and specialty details; otherwise, responses were anonymous. This project received a waiver, including a waiver of consent, from the institutional review board of the Memorial Sloan Kettering Cancer Institute, New York, New York. This study followed the AAPOR reporting guideline for web-based surveys.

Responses were evaluated with descriptive statistics (frequencies, cross-tabulations) and multivariable logistic regression, using 2-sided α = .05 to indicate statistical significance. Analyses were performed using R, version 3.6.3 (R Foundation for Statistical Computing).

## Results

Of 851 eligible oncologists, 346 (188 men, 158 women) completed the survey (41% response rate), and 342 answered all questions (99% completion rate). Table 1 lists respondent demographic characteristics.

**Table 1.** Physician Demographic Characteristics by Oncology Specialty

Characteristic	No. (%)					
	All	Medical oncology	Surgical oncology	Radiation oncology	Neuro-oncology	Other^a^
Baseline	346 (100)	118 (34)	52 (15)	46 (13)	7 (2)	123 (36)
Sex						
Male	188 (54)	62 (53)	33 (63)	30 (65)	3 (43)	61 (50)
Female	158 (46)	56 (47)	19 (37)	16 (35)	4 (57)	62 (50)
Years in practice						
≤5	112 (32)	48 (41)	10 (19)	24 (52)	4 (57)	26 (21)
6-25	165 (48)	53 (45)	25 (48)	18 (39)	2 (29)	67 (54)
>25	69 (20)	17 (14)	17 (33)	4 (9)	1 (14)	30 (24)

^a^ Other included pathologists, radiologists, anesthesiologists, and medical subspecialties that care for patients with cancer.

Most respondents (286 [83%]) believed that ways of preventing or mitigating patient vulnerability to financial toxicity exists and that financial toxicity is a potentially avoidable consequence of treatment, including 14 (5%) who believed they are completely preventable and 220 (77%) who believed they can be mitigated but not eliminated. Most believed they should play an active role in minimizing financial toxicity (285 [82%]), should be aware of patient vulnerability to financial toxicity before making treatment recommendations (270 [78%]), and could modify treatment plans to reduce costs for patients in need (239 [69%]). Most (232 [67%]) responded that they would modify their plans, of whom 56 (24%) would modify treatment dosing and frequency, 153 (66%) testing and imaging frequency, and 155 (67%) follow-up intervals. Most (221 [64%]) believed national guidelines should incorporate patient affordability concerns.

Only 76 respondents (22%) reported receiving any training on costs, affordability, or value-based care, with 4 (5%) reporting they received training on cost conversations, specifically.

In multivariable modeling to evaluate factors (sex, years in practice, specialty, training) associated with an oncologist’s willingness to change practice for high-risk patients, sex was found to be independently associated (Table 2). Female respondents were less likely than male colleagues to modify recommendations (odds ratio, 0.62; 95% CI, 0.38-0.99).

**Table 2.** Model of Willingness to Change Behavior in the Setting of High Risk of Financially Toxic Effects

Characteristic	No. (%)		Univariate *P* value	Multivariable odds ratio (95% CI)
	Willing to change behavior	Not willing to change behavior		
No. of respondents (N = 346)	232 (67)	114 (33)		
Sex				
Male	134 (71)	54 (29)	.08	1 [Reference]
Female	98 (62)	59 (38)		0.62 (0.38-0.99)
Years in practice				
≤5	76 (68)	36 (32)	.89	1.50 (0.76-2.94)
6-25	115 (70)	50 (30)		1.69 (0.92-3.11)
≥25	46 (67)	23 (33)		1 [Reference]
Specialty				
Medical oncology	78 (66)	40 (34)	.41	1.41 (0.71-2.81)
Surgical oncology	30 (58)	22 (42)		1 [Reference]
Radiation oncology	35 (76)	11 (24)		2.15 (0.87-5.29)
Neuro-oncology	5 (71)	2 (29)		1.96 (0.33-11.49)
Other^a^	84 (68)	39 (32)		1.63 (0.83-3.22)
Training on cost of care				
Yes	51 (67)	25 (33)	.99	0.98 (0.56-1.71)
No	181 (67)	89 (33)		1 [Reference]

^a^ Other included pathologists, radiologists, anesthesiologists, and medical subspecialties that care for patients with cancer.

## Discussion

In this survey study, the results reveal that oncologists at a major cancer center are sensitive to patient financial hardship and believe they should play an active role in minimizing financial harm. Previous research has revealed that most physicians receive minimal training in counseling patients about financial issues, despite interest.^3^ This finding is problematic because most patients prefer physicians who will discuss cost of care and because costs can be lowered through discussion with their physician.^6^

In cancer care delivery, the 2 parties making treatment decisions (oncologist and patient) rarely have insight into financial risks or incentives to choose the most cost-conscious options. Delivering high-quality care for each patient while containing costs poses an ethical challenge; we hope that equitable delivery of high-value care will improve with better price transparency, education, and physician awareness.

Limitations of this study include surveying oncologists at only 1 institution and respondents’ potential social desirability bias associated with affordability concerns. Nonetheless, the findings reveal that oncologists care about the patient’s financial burden of cancer treatment and believe they play a role in reducing that harm. Additional study is needed on the role of universal patient screening for vulnerability to financial toxicity and on multilevel interventions to reduce hardship.
